# PTSD, a Disorder with an Immunological Component

**DOI:** 10.3389/fimmu.2016.00219

**Published:** 2016-06-06

**Authors:** Zhewu Wang, M. Rita I. Young

**Affiliations:** ^1^Mental Health Service, Ralph H. Johnson VA Medical Center, Charleston, SC, USA; ^2^Department of Psychiatry, Medical University of South Carolina, Charleston, SC, USA; ^3^Research Service, Ralph H. Johnson VA Medical Center, Charleston, SC, USA; ^4^Department of Otolaryngology – Head and Neck Surgery, Medical University of South Carolina, Charleston, SC, USA

**Keywords:** cytokines, immune regulation, inflammation, plasma, post-traumatic stress disorder

## Abstract

Post-traumatic stress disorder (PTSD) has been associated with an inflammatory state. However, few studies have addressed the mechanisms underlying this immune imbalance that favors inflammation or how this imbalance contributes to PTSD. Whether the immune imbalance influences responsiveness or unresponsiveness of patients to PTSD treatments is currently not known. This review brings forward an immune emphasis to a mental health disorder that is unprecedented in its prevalence among combat Veterans of the ongoing conflicts in Iraq and Afghanistan and which also afflicts civilians who have undergone extreme traumatic experiences, such as following natural disasters, serious accidents, or assaults. Included is an overview of the correlative associations in human subjects between PTSD and inflammation and studies in animal models of PTSD, demonstrating causal contributions of inflammation and immune dysregulation to PTSD-like behavior following stress exposure.

## Introduction

Over 2.5 million U.S. service members have been deployed at Operation Enduring Freedom/Operation Iraqi Freedom (OEF/OIF) arenas since 2001, with an unprecedented proportion (15.7%) of them developing post-traumatic stress disorder (PTSD) following combat exposure (1, 2). The disorder is not limited to combat but is also seen in civilians following natural disasters, sexual trauma, or loss of family members (3, 4). PTSD is a debilitating disorder that develops after exposure to traumatic events. It is characterized by clusters of symptoms including re-experiencing the traumatic event, avoidance of trauma-related stimuli, general changes in mood and cognition, and hyperarousal symptoms. PTSD has a social and an economic impact on the afflicted individual and the community at large, and these affects can be long term. For example, 23 years after an earthquake experience, survivors exhibited PTSD anxiety symptoms and reduced quality of life (5). PTSD and depression were shown to worsen over time (1 year) following a major hurricane, with a doubling of the proportion of affected individuals with suicidal thoughts (6). The most comprehensive analysis of the impact of PTSD has been conducted with Veterans, but it has been suggested that the financial impact also applies to the civilian population with PTSD (7). Veterans with conflict-related PTSD and non-Veterans with PTSD have increased rates of substance use, other comorbidities, suicide, homelessness, unemployment, and reduced quality of life (2, 7, 8). A cost analysis showed that, of the high-cost patients to the VA health-care system, 17% were due to PTSD, and their high cost was attributed to increase number of inpatient stays, increased length of stay, and increased outpatient visits (9).

In the past two decades, psychological and pharmacological treatments have been developed for PTSD, but many patients do not respond to these treatments, placing them at risk for developing a chronic illness with poor long-term outcomes (10). Pharmacological treatment of Veterans with PTSD is limited by there being only two FDA-approved drugs for PTSD: sertraline and paroxetine. Both sertraline and paroxetine are in the class of selective serotonin reuptake inhibitors (SSRIs). Unfortunately, responsiveness to these first-line pharmacotherapies is suboptimal at 50–60% (11–14). Unknown is why combat Veterans appear to be more resistant to treatment with SSRIs than civilians (15). Also unclear is why some individuals respond to SSRIs and others do not. While only approximately half of the Veterans with PTSD respond to these first-line SSRIs, full remission is only 20–30% (11–14). Since there are no biomarkers to predict responsiveness to SSRI treatment, this leaves many PTSD subjects with the financial and emotional cost of ineffective treatments.

In the past decade, there has been an effort to identify the biological causes of PTSD. This effort has suggested that the biological dysfunction can result from immune alterations associated with PTSD (16), but relatively few studies have investigated immune functions in PTSD patients, and the results have been both unclear and contradictory. However, a compilation of the results indicates the presence of an excessive inflammatory state in subjects with PTSD (17–25), although the precise mechanisms of pathogenesis of PTSD remain unclear.

## Correlations between Inflammation and PTSD

Studies involving human subjects with PTSD have indicated a linkage between immune alterations and PTSD following trauma exposure, though it is unlikely that the primary cause of PTSD is immune-mediated. Measurement of the various immune mediators including pro-inflammatory cytokines in PTSD subjects has been somewhat haphazard, but collectively, the results of these studies have suggested immune alterations toward increased levels of inflammatory cytokines (4, 18, 19, 24, 26). An overview of these studies is in Table 1. These analyses have shown skewing toward increases in levels of pro-inflammatory cytokines, such as IL-1, IL-6, and TNF-α (4, 18, 19, 22, 24, 26). While levels of anti-inflammatory cytokines have been less frequently measured in subjects with PTSD, there have been some studies indicating reduced levels of these anti-inflammatory mediators, including IL-4 and TGF-β (19, 26). However, in a separate study, both inflammatory and anti-inflammatory cytokines were shown to be elevated in PTSD subjects, leading to a conclusion of a generalized immune activated state in PTSD (27).

**Table 1:** **Overview of studies with human subjects with PTSD**.

**Immune parameter**	**Pro-inflammatory mediators**	**Anti-inflammatory mediators**
Imbalance in blood cytokines to favor inflammation	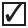 Increased inflammatory cytokines IL-1, IL-6, and TNF-α	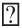 Reduced anti-inflammatory cytokines IL-4 and TGF-β
Skewing of peripheral blood cells populations	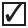 Increased pro-inflammatory Th1 and Th17	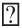 Decreases in anti-inflammatory regulators Treg and CD4^+^PD-L1^+^ cells
Increased inflammatory mediator in saliva	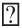 Increased MCP-1	
Effect of treatment on immune imbalance	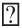 Reduced IL-1 and TNF-α	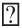 Restoration of Treg levels

*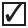 Strong experimental support; 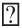 some support, but more studies are needed*.

The imbalances in cytokines in subjects with PTSD are reflected not only by increased plasma levels of inflammatory cytokines but also by imbalances in immune cell compositions. This is seen by increases in peripheral blood levels of pro-inflammatory Th1 and Th17 cells and reduced numbers of anti-inflammatory cells, such as Treg (18, 22). Levels of the negative regulator PD-L1 on CD4^+^ cells from PTSD patients are also reduced, further illustrating the immune skewing toward a dysregulated state (28).

Consistent with the pro-inflammatory skewing seen in subjects with PTSD is an increased incidence of allergies in this population. For example, the incidence of adult-onset asthma was shown to be increased subsequent to PTSD-associated trauma (29). A comparison between study participants with or without chronic idiopathic urticaria (CIU) showed the severity of PTSD to be greater in those with CIU, although the sequence of the appearance of the comorbidities was not determined (30). A retrospective study of Iraq and Afghanistan Veterans who were enrolled in the VA health-care system showed that those with PTSD had an increase in incidence of subsequently developing autoimmune diseases, such as thyroiditis, inflammatory bowel disease, multiple sclerosis, rheumatoid arthritis, and systemic lupus erythematosus (31). Interestingly, the reverse was not seen. Those with preexisting autoimmune disorders did not have an increased incidence of PTSD, indicating the severity of the immune imbalance following the traumatic episode.

Because of the direct association between skewed immune reactivity toward increased levels of inflammatory cytokines and disease severity in combat-exposed Veterans with PTSD, it has been suggested that immune activation is a contributor to their clinical status (20). A prospective study of deployed Marines showed that pre-deployment levels of C-reactive protein, an indicator of inflammation, predicted post-deployment PTSD and suggested inflammation to be a contributor to PTSD (32). A study of hospitalized patients with traumatic orthopedic injuries showed that those who subsequently developed PTSD had increased blood levels of inflammatory mediators and reduced anti-inflammatory mediators and suggested that this could be used as a biomarker for PTSD (19). However, at present, these suggestions may be premature as biomarkers for PTSD have yet to be clearly defined.

Assessments of inflammatory cytokine levels in the cerebral spinal fluid of subjects with and without PTSD have not been extensively measured. However, the results of such studies are conflicting, with demonstrations of both increases and no difference in levels of IL-6 among subjects with and without PTSD (33, 34). In contrast, studies have also shown increased levels of inflammatory mediators in saliva following stress exposure, although such studies of salivary cytokine levels are very few (3, 35). In one of these few studies, levels of the inflammatory mediator monocyte chemotactic protein (MCP-1) in the saliva of hurricane survivors with PTSD correlated with PTSD severity (3). It has previously been suggested that levels of immune mediators in the saliva can be influenced by sympathetic nervous system innervation, rather than being secondary to blood levels of the mediators (35, 36). This suggestion comes from demonstrations of earlier appearance of inflammatory cytokines in saliva following an acute stress than the increases that were seen systemically. Thus, salivary cytokine levels may be more reflective of mental health status than would plasma cytokine levels, but there has as yet not been sufficient studies to make definitive conclusions.

The plasticity of immune cells is driven by the cytokine milieu and raises the question of whether the inflammation-skewed immune reactivity of PTSD patients can undergo rebalancing. While studies have shown an imbalance toward inflammatory immune reactivity in PTSD patients, less clear is whether recovery from PTSD restores the immune balance. In fact, there are surprisingly few studies examining if clinical improvements of PTSD are accompanied by immune normalization. In one such study, women with PTSD had elevated levels of inflammatory cytokines, but those who recovered from PTSD had reduced levels of inflammatory cytokines that were similar to levels in healthy women who had not experienced trauma (37). Also suggesting immune rebalancing is demonstration of a shift from reduced levels of anti-inflammatory Treg cells in PTSD patients toward increased Treg levels upon narrative exposure therapy (17). Levels of the inflammatory cytokines IL-1 and TNF-α were reduced, and mental parameters improved following treatment of Veterans with a fermented soy product to normalize the blood steroidal hormone cascade (38).

Modulation of serotonin can directly impact immune activity (39–42), and thus, it is not unexpected that the SSRIs approved by the FDA for PTSD would have an impact on immune function. For example, blood levels of the inflammatory mediator IL-1 declined from an increased level in individuals with PTSD to levels comparable to those of controls following treatment with sertraline (43). Studies in which parameters of inflammation were measured prior to or after SSRI treatment for PTSD are relatively rare, but such studies have been conducted with SSRI treatment for other conditions. For example, children and adolescents who were treated with the SSRI, fluoxetine, for major depressive disorder or anxiety disorders had a decline in TNF-α levels, although not in levels of IL-6 or IL-1 (44). However, all three of these inflammatory cytokines were higher in subjects who were clinically resistant to SSRI treatment compared to those who responded to SSRI treatment. While the mechanisms for the immune quenching have not been fully defined, peripheral blood components, such as lymphocytes, monocytes, and platelets, contain serotonin. Inhibition of serotonin transporter by SSRIs may allow increased uptake through serotonin receptors, thereby decreasing immune function (41, 45, 46). Alternatively, studies have suggested that SSRIs mediate the immune inhibitory effects independently of serotonin transport by depletion of calcium stores of T-cells (47). Regardless of the mechanisms by which SSRIs mediate immune tempering, treatment of individuals having PTSD could impact not only clinical mental status but also immune balances. However, this needs to be validated with clinical intervention studies monitoring both clinical status and, concurrently, immune status. Also needed is analysis of immune status in PTSD subjects who are responsive to either psychotherapy or pharmacotherapy for PTSD versus those who resist treatment responses.

## Animal Models of PTSD Support a Causal Immune Role in PTSD

Studies with animal models have clearly established that the immune–brain communications are intimately intertwined. For example, immune mediators, such as IL-1, IL-6, and TNF-α, are able to cross the blood–brain barrier, and this is one way in which peripheral immune reactivity can have CNS impacts (48). Induction of peripheral inflammation by intraperitoneal injection of mice with endotoxin has been shown to trigger neuroinflammation in the CNS (49–51). This peripheral inflammation results in a concomitant increase in the number of activated microglia and in levels of inflammatory mediators IL-1, IL-6, and TNF-α in the hippocampus and in anxiety-like behaviors.

While animal models for PTSD are imperfect and unable to fully mimic PTSD in human subjects, studies using such animal models have been more comprehensive and definitive than studies of PTSD in human subjects. Several animal models for PTSD have been established and well characterized (52). An overview of the results of studies with animal models for PTSD is in Table 2. A commonly used model involves repeated exposure to either the rodent’s natural predator (typically cat) or urine of the predator. Also used are social stressors such as social housing instability induced by daily changes in cage cohorts or social defeat such as that induced by repeatedly exposing male mouse cohorts to an aggressive intruder mouse. Some studies have used combinations of predator exposure with social stressors. A stress-enhanced fear learning model involves unpredictable repeated shocks in one environment followed by a single shock in a second environment.

**Table 2:** **Overview of studies with animal models of PTSD that provide a causal immune–PTSD association**.

**Trigger**	**Immune response**	**Causality**
Severe stressor	Increased brain IL-1	Blocking IL-1 prevents fear response
Induction of peripheral inflammation	Increased brain levels of inflammatory cytokines IL-1, IL-6, and TNF-α	Block NF-κB reduces IL-1 and anxiety
Social defeat stress	Monocyte trafficking from spleen to brain	Splenectomy prevents monocyte trafficking and anxiety

Using predator exposure together with social stress of daily cage cohort change as a rat PTSD model, it has been shown that the dysregulation of cytokines that is seen peripherally in human subjects with PTSD is also seen in select brain regions where levels of the inflammatory cytokines are increased, while levels of the anti-inflammatory cytokines are reduced (19, 26, 53). Using a stress-enhanced fear learning model, hippocampal IL-1 levels were shown to become increased as a result of the severe stressor (54). This study further showed that blocking the IL-1 signaling pathway after the stress exposure prevented the fear learning response, suggesting that IL-1 could have a causal role in PTSD following trauma exposure. Also supporting immune contribution to PTSD-like anxiety is demonstration that anxiety in predator scent-stressed mice could be induced by activation of the pro-inflammatory NF-κB pathway, which is also increased in activity in human subjects with PTSD (55, 56). Interruption of this stress-provoked pro-inflammatory NF-κB pathway by toll-like receptor 9 (TLR9) activation reduced the elevated IL-1 levels and tempered the anxiety levels in the predator-stressed mice (55). What appears to be emerging is a critical involvement of the NF-κB inflammatory pathway in the anxiety of stress-conditioned animals. In fact, treatment of predator scent-stressed rats with either corticosterone or a selective NF-κB inhibitor mitigated stress behavior (57). This peripheral-CNS exchange is not limited to communication *via* soluble inflammatory mediators. In mice that were exposed to repeated social defeat, subthreshold stress resulted in trafficking of primed monocytes from the spleen to the brain and re-establishment of anxiety behavior (58, 59). However, if mice were splenectomized prior to the repeated social defeat, the monocyte trafficking and anxiety behavior, that otherwise occur following subthreshold stress, were prevented.

Similar to the results of the few studies that have been conducted to assess the impact of SSRIs on inflammatory mediators in human subjects (43, 44), studies with a predator-exposure animal model for PTSD showed that pharmacotherapy with the SSRI, sertraline, lessened the levels of the pro-inflammatory mediator IL-1 and signaling through the pro-inflammatory receptor TLR4 in the CNS, while increasing the CNS levels of anti-inflammatory cytokines IL-4 and IL-10 (53). The anxiety behavior and inflammation in the peripheral blood and CNS that is induced by treatment of rats with IFN-α could be blocked by treatment with the SSRI, paroxetine (60). However, as indicated above, serotonin can have direct immune modulating effects (41, 45, 46), raising the question of whether or not the alleviation of anxiety behavior in SSRI-treated animals could be secondary to the immunological effects of SSRIs.

## Challenges and Unknowns

A significant challenge to studying the immune association with PTSD is the variability in the type and severity of trauma exposure that led to PTSD. More conclusive results could be attained with a tightly controlled and relatively homogeneous population of subjects, with some having PTSD and others not. One such population could be Veterans of the currently ongoing conflicts in Iraq and Afghanistan, who are likely to be otherwise relatively healthy. However, such highly focused and tightly controlled studies would have to lead to studies that would be applicable to a broader population with PTSD. In studies with either a tightly controlled homogeneous population or a broader population with PTSD, it would be critical to measure both pro-inflammatory and anti-inflammatory cytokines and immune inflammatory and anti-inflammatory cells since immune reactivity consists of a balance between pro-inflammatory and anti-inflammatory forces. While studies have measured select pro-inflammatory cytokines, very few have also examined the balance between pro-inflammatory and anti-inflammatory mediators in subjects with PTSD.

Previously conducted studies that have suggested PTSD to be associated with an inflammatory state have traditionally measured levels of inflammatory cytokines in the blood plasma. However, analyses of cytokines in subjects undergoing other forms of stress exposure have shown more prominent immune alterations in saliva as opposed to plasma (35, 36). Collection of salivary samples for cytokine analysis is less invasive than collection of blood, and saliva can be collected more frequently, such as before, during, and after therapy sessions (3, 4, 61). In future studies, analysis of pro-inflammatory and anti-inflammatory cytokines in saliva of PTSD subjects will also need to be analyzed in the context of the subjects’ clinical status so as to further determine if the immune imbalances are more pronounced in subjects with more severe PTSD and whether the immune balances are restored upon response to therapy for PTSD.

More recent approaches to studying PTSD in humans have included advanced neuroimaging approaches and genetic approaches. For example, magnetic resonance images showed brain structural differences between subjects with and without PTSD, including differences in cortical thickness, volumes of caudate and right hippocampus, and total cerebral volume (62). Diffusion tensor imaging showed differences in measures of brain connectivity between subjects with and without PTSD (63). These studies have not, as of yet, incorporated the immune status of the subject with PTSD. In contrast, genetic analyses for predictors of PTSD risk have implicated immune genes as biomarkers. A study of pre-deployment military showed that service members who eventually developed PTSD had dysregulated expression of immune-associated genes, which could be used to predict who subsequently develops PTSD (64). However, such results should not come as a surprise as they reflect at the genetic level the immune dysregulation that has been shown at the protein level. However, such studies are starting to address the molecular mechanisms that could be underlying the immune skewing toward a dysregulated inflammatory state in PTSD (65).

The underwhelming level of responsiveness to treatments for PTSD urges the exploration of alternate treatment. If immune–brain communications are contributors to PTSD following the initial trauma, then this opens the possibility of immune intervention. However, immune modulatory treatment approaches need to be accompanied by mechanistic studies to define the relationships in PTSD between behavior and peripheral and CNS immune reactivities. A key question is whether treatment effectiveness with SSRIs is a consequence of immune modulation and whether immunological mechanisms bear responsibility when SSRI treatment is ineffective. Alternate treatments being tested include magnetic brain stimulation (66), but the immunological impacts have not been determined. Despite these unanswered questions, the culmination of studies from subjects with PTSD and from animal models shows that PTSD is not only a behavioral disorder but is also an immunological disorder with immune imbalances toward an increased pro-inflammatory state and diminished anti-inflammatory controls.

## Author Contributions

This manuscript was written by MY with close collaboration and contribution by ZW.

## Conflict of Interest Statement

The authors declare that the research was conducted in the absence of any commercial or financial relationships that could be construed as a potential conflict of interest.
